# Bonding in a Crystalline Tri‐Thorium Cluster: Not σ‐Aromatic But Still Unique

**DOI:** 10.1002/anie.202204337

**Published:** 2022-04-29

**Authors:** Dariusz W. Szczepanik

**Affiliations:** ^1^ Department of Theoretical Chemistry, Faculty of Chemistry Jagiellonian University Gronostajowa 2 30-387 Kraków Poland

**Keywords:** Actinide Bonding, Aromaticity, Charge-Shift Bonding, Chemical Resonance, Multicenter Bonding

## Abstract

Very recently, Boronski et al. reported the first thorium–thorium bond in a crystalline cluster prepared under normal experimental conditions. By using a range of experimental techniques and computational models, the authors found that the isolated actinide cluster contains at its heart two paired electrons delocalized over the tri‐thorium ring. The recorded Raman spectrum allegedly confirmed the existence of a σ‐aromatic three‐center two‐electron bond. In the following we demonstrate that the experimentally observed broad inelastic scattering bands between 60 and 135 cm^−1^, originally assigned by the authors to thorium‐thorium vibrations, represent the combination of Th−Cl stretching and Th−Cl−Th bending modes, and they establish the existence of an unprecedented multicenter charge‐shift bonding (ThCl_2_)_3_ rather than the σ‐aromatic bonding Th_3_. In the light of the presented findings, the latter remains experimentally unproven and computationally questionable.

## Graphical abstract

High symmetry and unusual thermodynamic stability of the recently prepared and isolated crystalline tri‐thorium cluster is determined by the unique multicenter charge‐shift bonding pattern (ThCl_2_)_3_ rather than the σ‐aromatic Th_3_ bonding. The delocalized (ThCl_2_)_3_ bond extends the range of the charge‐shift bonding beyond transition metals to a record seventh row of the periodic table.

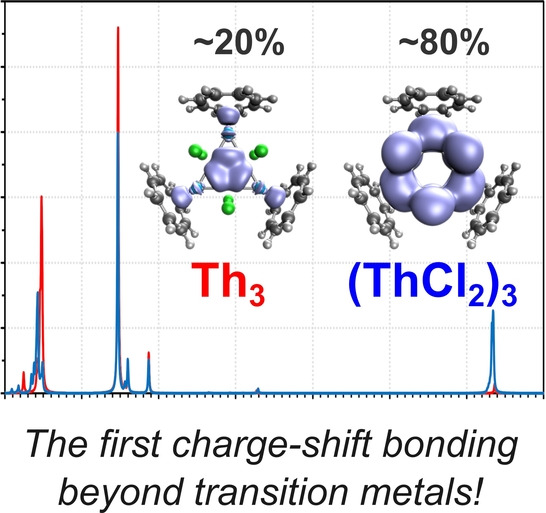

The experimental realization of actinide‐actinide bonding in isolable molecules has been one of the main targets of synthetic actinide chemistry for decades.^[1]^ Very recently, Boronski et al. reported the first thorium–thorium bonding in a crystalline cluster prepared and isolated under normal experimental conditions, [{Th(η^8^‐C_8_H_8_)(μ_3_‐Cl)_2_}_3_{K(THF)_2_}_2_]_∞_ (**3**).^[2]^ The electron paramagnetic resonance spectroscopy and the superconducting quantum‐interference device magnetometry revealed that **3** contains at its heart two paired electrons equally distributed over the symmetric (*D*_3*h*_) tri‐thorium ring, and the recorded Raman spectrum allegedly confirmed the existence of a three‐center two‐electron (3c–2e) bond. This type of delocalized σ‐bonding is very often referred to as σ‐aromatic bonding, and therefore this discovery has been acclaimed as extending the range of the aromatic stabilization effect to a record sixth principal atomic quantum shell and to the seventh row of the periodic table.^[2]^

To understand the electronic structure of **3**, the authors computationally investigated several model clusters, including [{Th(η^8^‐C_8_H_8_)(μ‐Cl)_2_}_3_K_2_] (**3′′**) with the Highest Occupied Molecular Orbital (HOMO) reminiscent of a 3c–2e bonding, and its dication [{Th(η^8^‐C_8_H_8_)(μ‐Cl)_2_}_3_K_2_]^2+^ (3*) without the 3c–2e HOMO. The authors found that the geometric structure of **3′′** better fits the X‐ray diffraction data and the calculated vibration modes match well with the intensive signals observed in the experimental Raman spectrum, which they interpreted as the *“experimental confirmation of the Th_3_ bonding in **3**”*.^[2]^ However, such an interpretation is mostly inaccurate as the same vibration modes feature the model cluster 3*, which in fact lacks any direct actinide–actinide bonding (indeed, the corresponding bond orders calculated for **3′′** and 3* are 0.373 and 0.005, respectively). In particular, as shown in Figure 1a, the characteristic modes centered at 70.5, 76.9 and 105.0 cm^−1^ in **3′′** can easily be found respectively at 63.6, 74.8 and 102.2 cm^−1^ in 3*, although the lack of delocalized and highly polarizable HOMO in the latter makes them hardly detectable in the Raman spectroscopy. Furthermore, the displacement vectors on thorium and chlorine atoms in both model clusters have comparable magnitudes, which strongly suggests that the observed broad inelastic scattering bands between 60 and 135 cm^−1^ represent collective stretching and bending modes of the actinide–halogen rather than actinide–actinide bonding.

**Figure 1:**
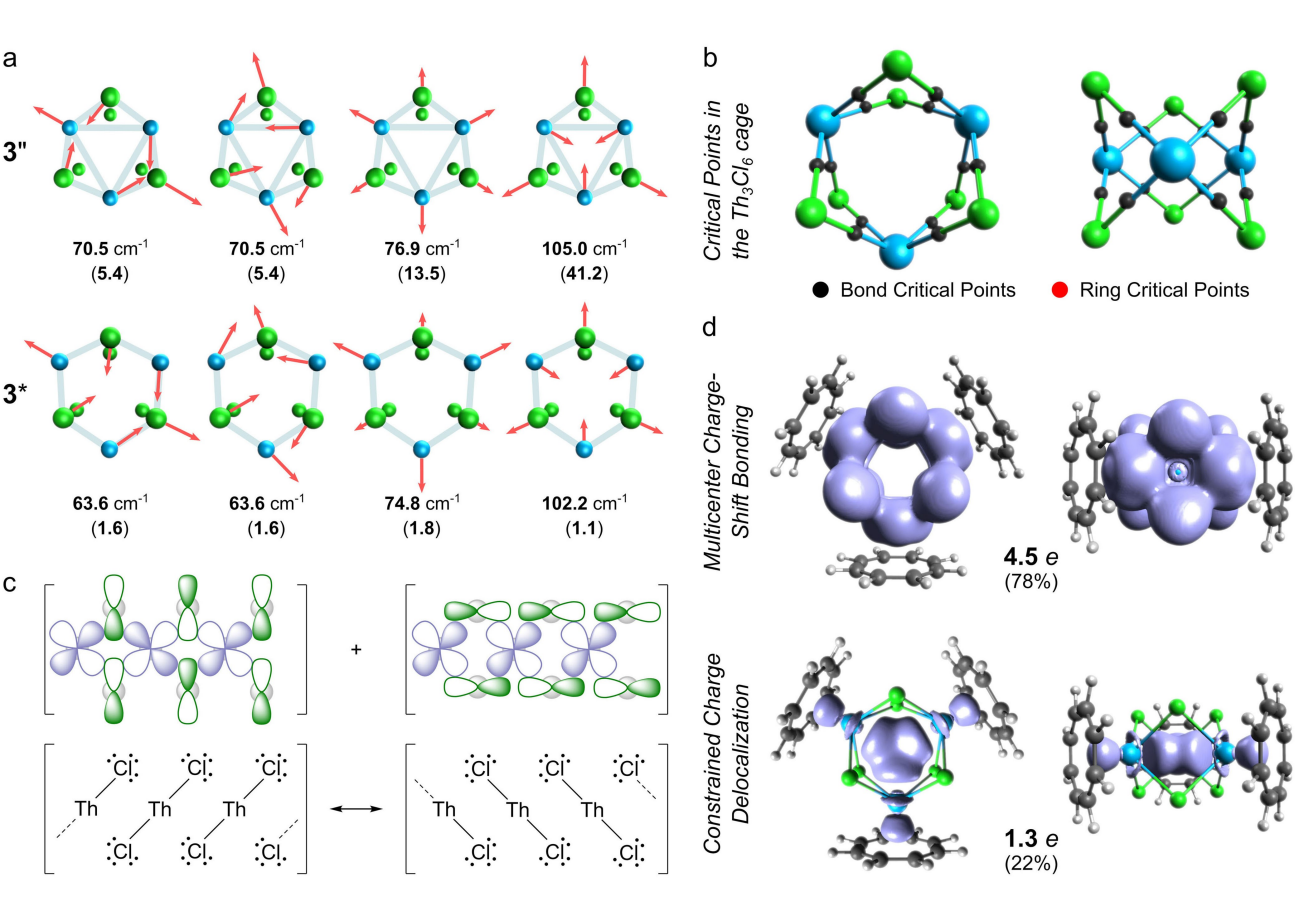
a) The selected vibration modes calculated for the model clusters **3′′** (neutral) and 3* (dication without the 3c–2e HOMO) with the corresponding Raman scattering activities in brackets in units A^4^AMU^−1^ (atoms outside the Th_3_Cl_6_ cage were excluded). b) Critical points from the analysis of the electron density calculated for the model cluster **3′′** (left and right columns depict top and side view of the Th_3_Cl_6_ fragment, respectively). c) The linearized side‐view of two orbital conjugation topologies in which the overlap of the 6d (Th) and 3p (Cl) atomic orbitals in the Th_3_Cl_6_ cage is particularly effective; below the resonance Lewis structures that rationalize the delocalized (“resonating”) nature of the thorium‐chlorine bonding in the Th_3_Cl_6_ cage in **3′′**. d) Isosurfaces (at 0.003*e*) and the corresponding electron populations of the EDDB function calculated for the Th_3_Cl_6_ cage (ignoring the metal–metal interactions) and the Th_3_ subunit in **3′′** (K atoms were excluded).

The dominant role of the Th−Cl bonding in the Th_3_Cl_6_ cage becomes particularly evident if one compares the overall Th_3_ bond order in **3′′**, that is 3×0.373=1.119, with twelve polarized thorium–chlorine bonds, 12×0.497=5.964; here the full bond order of 0.497 is a sum of the covalent component of 0.198 and the electrovalent (ionic) component of 0.299 estimated from the Evarestov–Veryazov equation.^[3]^ These numbers clearly show that about 84 % of the chemical interactions in the entire Th_3_Cl_6_ cage in the model cluster **3′′** are associated with the polarized thorium–chlorine bonds. The topological analysis of the one‐electron density calculated for the model cluster **3′′** fully confirms the existence of twelve polarized thorium–chlorine bonds (Figure 1b). However, the lack of bond and ring critical points that could be certainly attributed to the Th_3_ unit suggests that the thorium atoms may be not bonded at all. In this context, a small but noticeable value of the bond order (0.373) does not necessarily imply bonding character as it may result from the overlap of the extremely sized 6d orbitals being “squeezed” inside the Th_3_ unit due to the highly symmetric (*D*_3*h*_) arrangement of the thorium–chlorine bonds in the Th_3_Cl_6_ cage. In other words, the uniform charge distribution over three thorium atoms in **3′′** may have nothing to do with neither 3c–2e bonding nor the aromatic stabilization. Indeed, the additional analyses of the effect of the 3c–2e HOMO on thermodynamic stability (Figure S1) and orbital energy levels of the remaining occupied molecular orbitals (Table S1 and Figures S2 and S3) only further confirm that the existence of the tri‐thorium bonding in the crystalline cluster **3** is computationally questionable.

It should be mentioned that the reported by the original authors remarkable negative values of the nucleus‐independent chemical shifts (NICS),^[4]^ suggesting aromaticity, are meaningless as NICS is principally unable to distinguish between the magnetic shielding caused by the aromatic ring currents and the local circulations from the surrounding chemical bonds and/or lone‐pairs.[^5a^, ^5b^, ^5c^] Very recently, Cuyacot et al.^[5d]^ have demonstrated that NICS indeed fails in this particular case, and the magnitude of the magnetically‐induced paratropic ring current found inside the Th_3_ unit in **3′′** is marginal, which is exactly what one would expect for either an ordinary non‐aromatic ring or a non‐bonded system.

The experimental Raman spectrum recorded by Lidle and co‐workers and the computational data and arguments presented above indicate that the unusual actinide–halogen bonding pattern is a vital factor determining the high symmetry and stability of the actinide cluster **3**. To elucidate the nature of bonding in the Th_3_Cl_6_ cage, different orbital conjugation topologies has been considered involving the atomic orbitals 6d (thorium) and 3p (chlorine). Two configurations, in which the in‐phase orbital overlap is particularly effective are presented in Figure 1c. Admittedly, each pair of thorium atoms does not sit in the same plane with the chlorine atoms, but due to extreme size of the 6d orbitals (which easily penetrate the van der Waals spheres of chlorine atoms) the orbital overlap is still expected to be very effective. The superposition of these two configurations enables a resonance mode with the lone‐pairs and the polarized thorium–chlorine bonds cooperatively switch their positions; such bonding motif resembles the charge‐shift bonding known from the literature.^[6]^ The resonance of two possible Lewis structures depicted in Figure 1c gives rise to a formally half‐bond between thorium and chlorine atoms, which perfectly agrees with the previously calculated full bond order of 0.497. Furthermore, the calculated full chemical valencies for the thorium (4.010) and chlorine (1.006) atoms are very close to the corresponding formal valencies of IV and I, respectively. Since each thorium atom incorporates two electrons to aromatize the cyclooctatetraene ligands (see Figure S4), the thorium and chlorine atoms within the Th_3_Cl_6_ cage clearly act as divalent and monovalent elements, respectively, which again perfectly corresponds to the resonance Lewis structures representing the delocalized actinide–halogen charge‐shift bonding (ThCl_2_)_3_ (Figure 1c). To confirm the delocalized (“resonating”) character of bonding in the Th_3_Cl_6_ cage in **3′′**, the electron density of delocalized bonds (EDDB) method has been used,^[7]^ which provides a unique capability to extract from the calculated electron density the part (a density “layer”) that represents strictly the electrons delocalized between different bond positions. The resulting EDDB contours and the corresponding electron populations presented in Figure 1d provides very clear and distinct picture of delocalization in the Th_3_Cl_6_ cage that in 78 % is determined by the multicenter charge‐shift bonding (ThCl_2_)_3_. Interestingly, the characteristic shape of the EDDB function in the Th_3_ core reveals noticeable reduction of the electron density in the centroid and the bond midpoints, which explains the lack of the ring and bond critical points and reaffirms again that the charge delocalization over the thorium atoms may have nothing to do with neither the three‐center σ‐bonding nor the σ‐aromatic stabilization.

To summarize, the presented analysis clarifies inaccurate conclusions of the original study by Boronski et al.,^[2]^ and reveals that the high symmetry (*D*_3*h*_) and unusual thermodynamic stability of the crystalline tri‐thorium cluster **3** is mostly determined by the unprecedented multicenter charge‐shift bonding (ThCl_2_)_3_ rather than the σ‐aromatic Th_3_ bond. In the light of the presented findings, the existence of the latter remains experimentally unproven and computationally questionable, and if the tri‐thorium bonding exists at all, it should not be expected to be stronger than the extremely weak Th−Th bonds already reported in the literature.^[1]^ In contrast, the charge‐shift metal–halogen bonding has recently been shown to be particularly strong when 5d orbitals of the transition‐metals from Groups 11 and 12 are involved.^[6c]^ Accordingly, the thorium atom is also expected to form bonds of this type due to strong Pauli repulsion between the valence 6d^[2]^ and 7s^[2]^ electron pairs. Therefore, the unique thorium–chlorine bonding pattern (ThCl_2_)_3_ found in the crystalline cluster **3** extends the range of the charge‐shift bonding beyond transition metals to a record seventh row of the periodic table. This discovery may have broader implications for understanding the chemistry of actinides and future attempts to design and synthesize new stable actinide complexes.

## Conflict of interest

The authors declare no conflict of interest.

## Supporting information

As a service to our authors and readers, this journal provides supporting information supplied by the authors. Such materials are peer reviewed and may be re‐organized for online delivery, but are not copy‐edited or typeset. Technical support issues arising from supporting information (other than missing files) should be addressed to the authors.

Supporting Information

## Data Availability

Computational details and the data that support the findings of this study (additional figures, tables, optimized geometry coordinates, basis‐set specification, etc.) are available in the Supporting Information of this article.
