# Supplementary material for: Bonding in a Crystalline Tri‐Thorium Cluster: Not σ‐Aromatic But Still Unique
Source: Angew Chem Int Ed Engl. 2022 Apr 29;61(27):e202204337. doi: 10.1002/anie.202204337 (PMC9325075; doi:10.1002/anie.202204337)
Supplement: Supplementary file 1 — Supporting Information [file ANIE-61-0-s001.pdf]

Angewandte Chemie
Eine Zeitschrift der Gesellschaft Deutscher Chemiker

Supporting Information

Bonding in a Crystalline Tri-Thorium Cluster: Not σ-Aromatic But Still Unique

*D. W. Szczepanik**

Computational Details

Molecular geometry optimization and the harmonic vibrational frequency calculations (to confirm minima, simulate infrared and Raman spectra, and to determine thermochemical parameters) were performed with Gaussian G16 (revision C01).[1] The same (validated) level of the theory was used as in the original paper by Liddle and co-workers,[2] i.e., the density functional theory (DFT) with the exchange-correlation (XC) functional PBE0,[3] the standard cc-pVTZ basis set for carbon and chlorine atoms, cc-pVDZ for hydrogen atoms, 6-311G* for potassium and/or argon atoms, the small-core Stuttgart-Bonn relativistic effective-core potential (replacing 60 core electrons) and the associated segmented valence basis sets for thorium,[4-6] and Grimme's D3 dispersion corrections[7] with the Becke-Johnson damping.[8-10] SuperFine grid and tight convergence criteria were used in all the self-consistent field (SCF) calculations. To improve the molecular geometry optimization process, initial optimization was performed without constrains using the Rational Function Optimization approach,[11] and the exact force constants were calculated at every optimization step. The final geometries were obtained by re-optimization of the initially preoptimized geometries with tight convergence criteria and the symmetry constraints (D_{3h}) applied to geometry, density and Fockian matrices and electron integrals. All the wavefunctions were confirmed to be ground-state minima with stable closed-shell configurations by the procedure implemented in Gaussian (the keyword "Stable").[1] The results of the vibrational analyses are presented in **Figure 1a** while the key structural and thermochemical parameters for the model clusters are given in **Figure S1**.

The molecular orbital (MO) analyses were carried out at the DFT level using the same XC functional as in the geometry optimization, i.e., PBE0. To address the long-range exchange effects on the Kohn-Sham orbital energy levels,[12] the long-range corrected version of the PBE0 functional as proposed by Henderson *et al.*,[13] i.e., LC-ωHPBE, was used in the single-point calculations.

Additionally, the relativistic effects were investigated in the Douglas-Kroll-Hess 4th-order (DKH4) LC-ωHPBE single-point calculations (including the spin-orbit coupling terms)[14-16] involving the polarized all-electron triple-zeta valence basis by Jorge and co-workers.[17] To qualitatively assess the potentially globally stabilizing/destabilizing effect of the 3c-2e HOMO orbital by comparison of the molecular orbital energies, a new neutral model **3**† has been proposed that is isoelectronic with **3***. The results of the MO analyses are collected in **Table S1** and **Figures S2** and **S3**.

The wavefunction analyses (covalent and ionic component of the bond orders, topological analysis of the electron density, etc.), as well as the Electron Density of Delocalized Bonds (EDDB) analysis[11] were carried out at the fully relativistic LC-ωHPBE (DKH4) level since the population analyses involving the ECP densities for such heavy atoms have been demonstrated to significantly underestimate the electrovalent character of the metal-halogen bonding.[19] The natural atomic charges and the covalent (quadratic) bond indices were calculated using the NBO7 program (version 7.0).[20] The quantum-chemical full bond orders and the corresponding full chemical valencies of atoms were calculated based on the modified Evarestov-Veryazov equation (the required parameters such as atomic charges and bond covalencies were taken from the NBO7 program).[21] The RunEDDB code (version 26-Jun-2021)[22] was used to perform the EDDB analysis. To assess delocalization in the Th_3Cl_6 cage, the $EDDB_E(r)$ function was used with all the parameters set to default. The electron delocalization in the Th_3 'ring' was quantified using the $EDDB_F(r)$ function. Topological analysis of the calculated fully relativistic one-electron density was performed using the MultiWFN program (version 3.8).[23] The critical points identifying bonds (+3,-1) and rings (+3,+1) were obtained from the standard searching procedure involving the midpoints of all the atomic pairs in the Th_3Cl_6 cage. The results of the topological and EDDB analyses are presented in **Figure 1b** and **1d** in the main body of the manuscript.

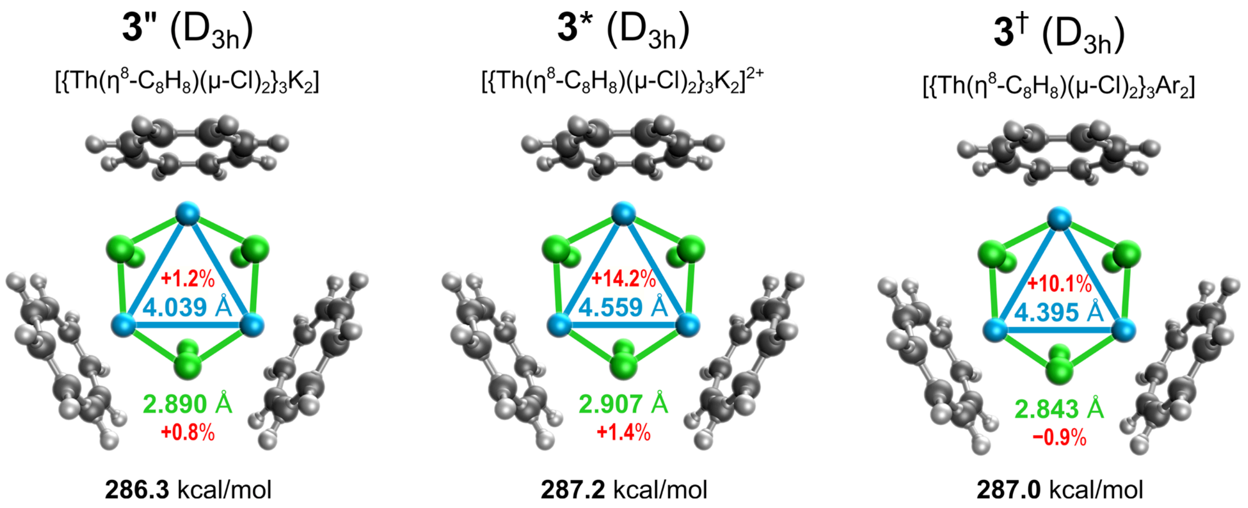

Figure S1. Calculated distances between thorium atoms (blue numbers) and the Th–Cl bond lengths (green numbers) in three different model tri-thorium clusters (potassium and/or argon atoms have been removed for clarity). Red numbers stand for the percentage deviations from the corresponding averaged experimental bond lengths of 3.991 Å (thorium-thorium) and 2.868 Å (thorium-chlorine). The vibrational contributions to the thermal energy are given below the molecular structures.

Table S1. Energies of five the lowest unoccupied and ten the highest occupied molecular orbitals in three model clusters calculated using both the effective core potential (ECP) as well as the fully relativistic DKH4 method (see Computational Details). Bold numbers refer to the orbital energies used in **Figure S2**.

MOs	**3”**			**3***			**$3^{\dagger}$**		
	PBE0 (ECP)	LC-ωHPBE (ECP)	LC-ωHPBE (DKH4)	PBE0 (ECP)	LC-ωHPBE (ECP)	LC-ωHPBE (DKH4)	PBE0 (ECP)	LC-ωHPBE (ECP)	LC-ωHPBE (DKH4)
LUMO+4	-0.42	0.82	1.79	-6.43	-4.21	-4.25	-1.26	0.92	2.07
LUMO+3	-0.70	0.74	1.31	-6.43	-4.21	-4.25	-1.26	0.92	2.07
LUMO+2	-0.70	0.74	1.31	-6.47	-4.31	-4.34	-1.34	0.76	0.87
LUMO+1	-1.04	-0.09	-0.06	-6.47	-4.43	-4.44	-1.34	0.76	0.87
LUMO	-1.15	-0.10	**-0.08**	-7.47	-5.41	**-4.89**	-2.38	-0.34	**0.02**
HOMO	-3.27	-5.15	**-4.76**	-10.99	-13.20	**-12.99**	-6.09	-8.29	**-8.10**
HOMO-1	-5.14	-7.28	**-7.13**	-11.12	-13.34	**-13.33**	-6.21	-8.43	**-8.43**
HOMO-2	-5.36	-7.53	**-7.50**	-11.12	-13.34	**-13.33**	-6.21	-8.43	**-8.43**
HOMO-3	-5.36	-7.53	**-7.50**	-11.14	-13.35	**-13.37**	-6.24	-8.44	**-8.46**
HOMO-4	-5.42	-7.57	**-7.56**	-11.19	-13.40	**-13.48**	-6.29	-8.50	**-8.60**
HOMO-5	-5.49	-7.65	**-7.70**	-11.19	-13.40	**-13.48**	-6.29	-8.50	**-8.60**
HOMO-6	-5.49	-7.65	**-7.70**	-13.94	-16.78	**-16.95**	-8.51	-11.25	**-11.39**
HOMO-7	-8.33	-11.12	**-11.39**	-13.94	-16.78	**-16.95**	-8.51	-11.25	**-11.39**
HOMO-8	-8.33	-11.12	**-11.39**	-13.95	-16.79	**-16.97**	-8.81	-11.55	**-11.72**
HOMO-9	-8.39	-11.20	**-11.49**	-14.05	-16.86	**16.97**	-8.89	-11.55	**-11.83**

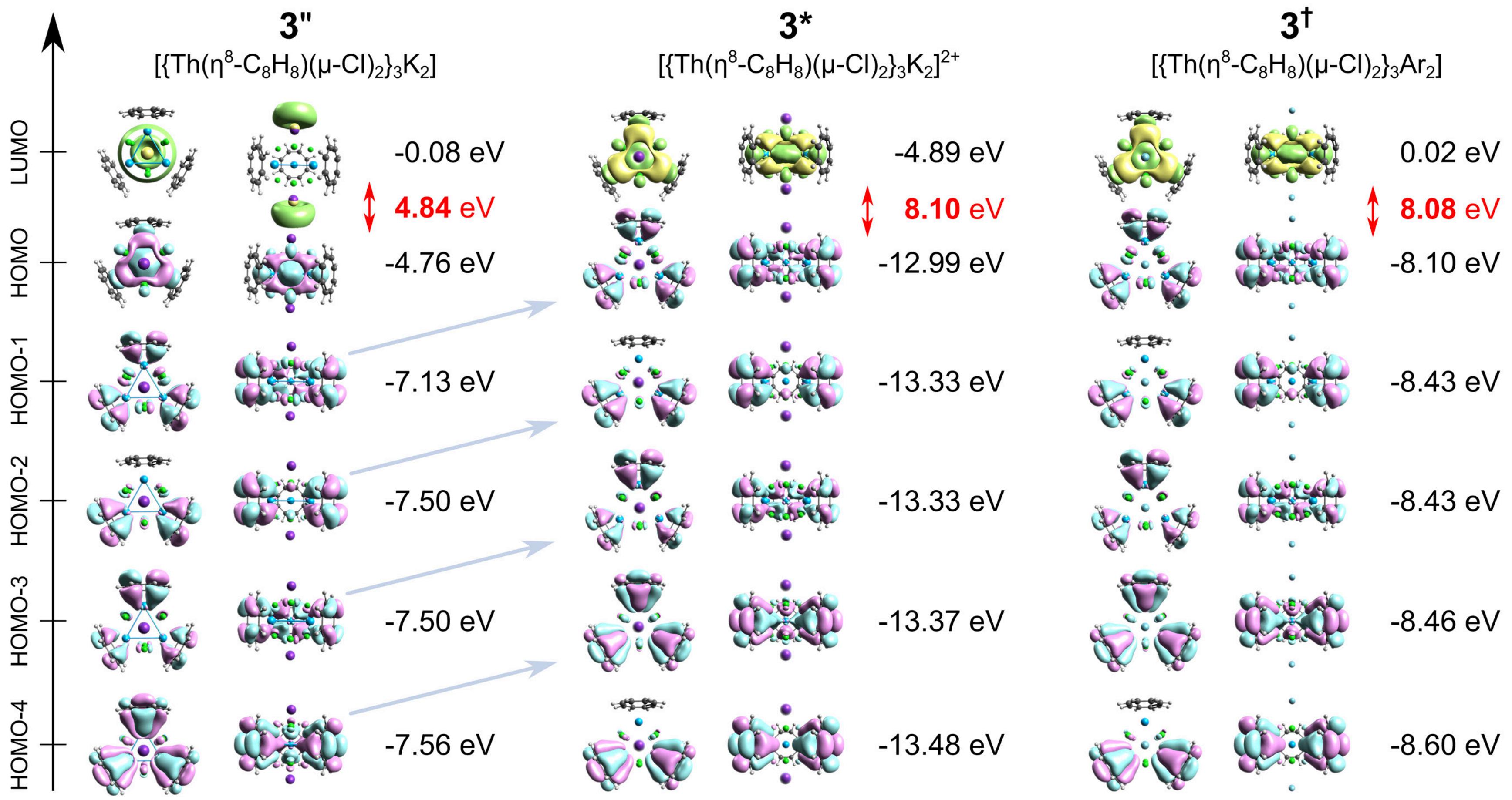

Figure S2. Kohn-Sham molecular orbitals with the corresponding orbitals energies from the fully relativistic calculations for three different model tri-thorium clusters. Red bold numbers correspond to the HOMO-LUMO gaps.

Comment: *The neutral model* ***3†*** *has almost the same HOMO-LUMO gap as the isoelectronic but charged* ***3****, *which enables direct comparison of the corresponding HOMO-n orbitals and their energies with the ones from the neutral model* ***3"***. *These numbers suggest that the presence of the 3c-2e HOMO in* ***3"*** *may have noticeably destabilizing character for the entire cluster.*

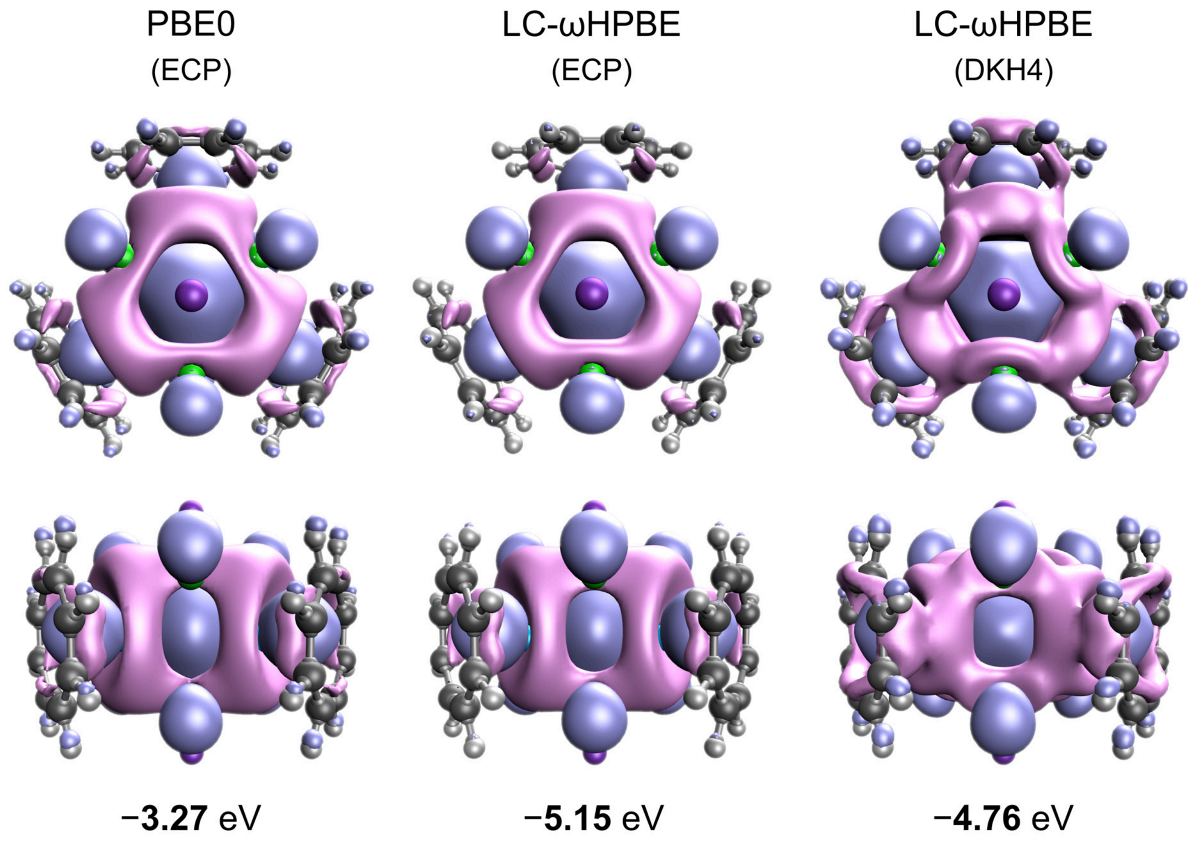

Figure S3. The highest occupied molecular orbitals in **3**" with the corresponding orbitals energies from the non-relativistic (ECP) and the fully relativistic calculations (DKH4).

Comment: *An inhomogeneous structure of HOMO is especially evident in the DHK4 case where about 20-25% of the molecular orbital population (according to the Mulliken condensed populations) comes from different atomic orbitals of the thorium, chlorine, carbon and even hydrogen atoms, suggesting their local (not global) stabilizing role in the chemical interactions involving thorium atoms.*

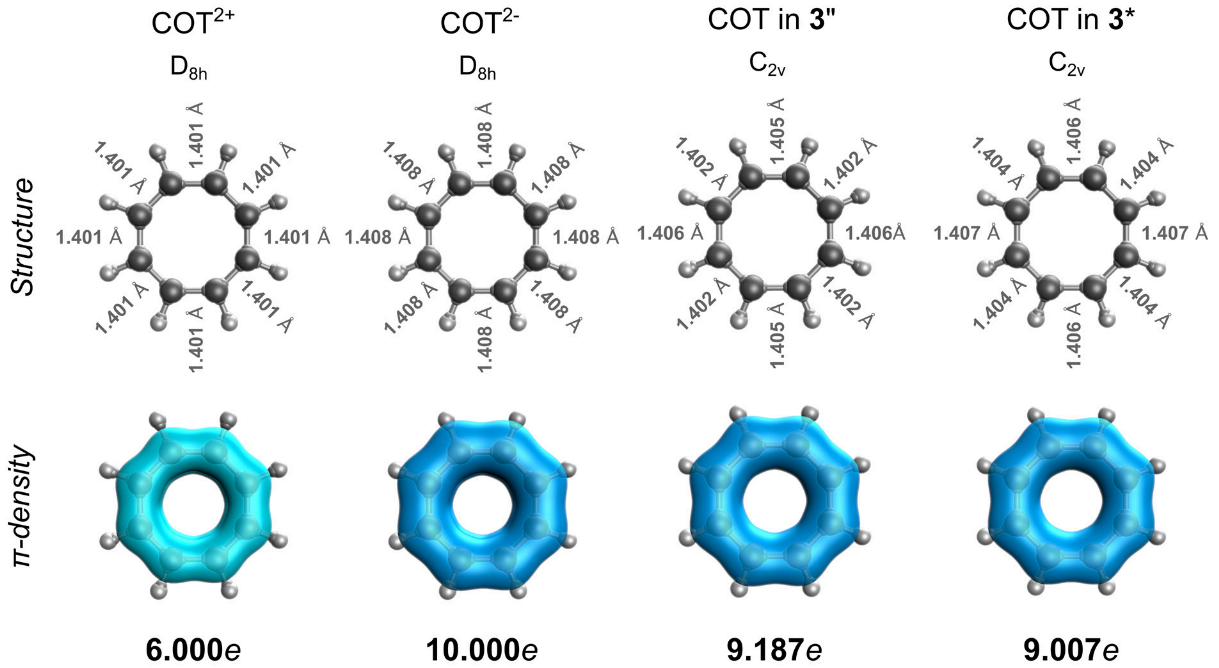

Figure S4. The structural and π-electronic characteristics of the cyclooctatetraene (COT) ligands in **3”** and **3*** (the remaining fragments of the molecule are excluded), and the isolated aromatic COT dication (at the planarized geometry) and dianion (at the ground-state minimum geometry). Bold numbers below the structures in the second row refer to the total population of π-electrons assigned to carbon atoms that were derived from the σ/π-dissected $EDDB_G$ population analysis.

Comment: *Despite noticeable distortions from the D_{8h} symmetry and the resulting non-integer population of π-electrons (partially shared with the metal atoms) it is evident that the COT ligands in both models **3”** (neutral) and **3*** (charged) should be classified as the Hückel's 10π-aromatics.*

Gaussian input files with the final (optimized) coordinates

3” (D_{3h}, *no imaginary frequencies*)

```
%Chk=3prime_pbe0.chk
# PBE1PBE/GenECP EmpiricalDispersion=GD3BJ Int(SuperFineGrid) SCF(Symm,DSymm,FSymm,IntRep) Symmetry(PG=D3h,Follow,On)
Opt(RFO,Tight,CalcAll,NoEigenTest) Freq(Raman)

3prime_pbe0

0 1
Th        0.000000003676     -2.331807846613      0.000000000000
Cl       -1.880468583210     -1.085689046845      1.807180476457
C        -0.703134306454     -4.348218002614      1.693235569867
H        -1.119098025522     -4.247228632650      2.692116569687
Th        2.019404829923      1.165903926231      0.000000000000
Cl        1.880468586634     -1.085689040915      1.807180476457
C        -1.694784041438     -4.346866505486      0.702572962135
H        -2.692896877035     -4.235028516834      1.115775995805
Cl        1.880468586634     -1.085689040915     -1.807180476457
C        -1.694784041438     -4.346866505486     -0.702572962135
H        -2.692896877035     -4.235028516834     -1.115775995805
C        -0.703134306454     -4.348218002614     -1.693235569867
H        -1.119098025522     -4.247228632650     -2.692116569687
C         0.703134320165     -4.348218000397     -1.693235569867
H         1.119098038915     -4.247228629121     -2.692116569687
C         1.694784055145     -4.346866500141     -0.702572962135
H         2.692896890389     -4.235028508343     -1.115775995805
C         1.694784055145     -4.346866500141      0.702572962135
H         2.692896890389     -4.235028508343      1.115775995805
C         0.703134320165     -4.348218000397      1.693235569867
H         1.119098038915     -4.247228629121      2.692116569687
C         3.414100089304      2.783041183474     -1.693235569867
H         3.118658868892      3.092781645326     -2.692116569687
C         4.611888841179      0.705707218669      0.702572962135
H         5.014090719698     -0.214602847127      1.115775995805
C         4.611888841179      0.705707218669     -0.702572962135
H         5.014090719698     -0.214602847127     -1.115775995805
C         4.117234404534      1.565176829386     -1.693235569867
H         4.237756904167      1.154446996638     -2.692116569687
C         4.117234404534      1.565176829386      1.693235569867
H         4.237756904167      1.154446996638      2.692116569687
C         2.917104788260      3.641159295495     -0.702572962135
H         2.321193828632      4.449631370761     -1.115775995805
C         2.917104788260      3.641159295495      0.702572962135
H         2.321193828632      4.449631370761      1.115775995805
C         3.414100089304      2.783041183474      1.693235569867
H         3.118658868892      3.092781645326      2.692116569687
Th       -2.019404833599      1.165903919863      0.000000000000
Cl       -1.880468583210     -1.085689046845     -1.807180476457
C        -4.117234409469      1.565176816403     -1.693235569867
H        -4.237756907807      1.154446983276     -2.692116569687
Cl       -0.000000003423      2.171378087241     -1.807180476457
C        -4.611888843405      0.705707204127     -0.702572962135
H        -5.014090719021     -0.214602862937     -1.115775995805
Cl       -0.000000003423      2.171378087241      1.807180476457
C        -4.611888843405      0.705707204127      0.702572962135
H        -5.014090719021     -0.214602862937      1.115775995805
C        -4.117234409469      1.565176816403      1.693235569867
H        -4.237756907807      1.154446983276      2.692116569687
C        -3.414100098080      2.783041172709      1.693235569867
H        -3.118658878645      3.092781635492      2.692116569687
C        -2.917104799741      3.641159286297      0.702572962135
H        -2.321193842663      4.449631363442      1.115775995805
C        -2.917104799741      3.641159286297     -0.702572962135
H        -2.321193842663      4.449631363442     -1.115775995805
C        -3.414100098080      2.783041172709     -1.693235569867
H        -3.118658878645      3.092781635492     -2.692116569687
K         0.000000000000     -0.000000000173     -3.818154929045
K         0.000000000000     -0.000000000173      3.818154929045

C Cl 0
cc-pVTZ
****
H 0
cc-pVDZ
****
K 0
6-311G*
****
Th     0
S    4   1.00
     10.5166570              0.0330960
      7.0109060             -0.2339080
      4.6738090              0.5198480
      1.8620810             -1.0189420
S    1   1.00
      0.5373270              1.0000000
S    1   1.00
```

```
      0.2614820              1.0000000
S    1   1.00
      0.0682570              1.0000000
S    1   1.00
      0.0275220              1.0000000
P    4   1.00
     15.8728110              0.0015590
     10.5818740             -0.0069170
      3.9249540              0.1150440
      2.2082920             -0.3349900
P    1   1.00
      0.4929470              1.0000000
P    1   1.00
      0.2151610              1.0000000
P    1   1.00
      0.0545080              1.0000000
P    1   1.00
      0.0143650              1.0000000
D    3   1.00
      6.0714120              0.0017310
      1.9840490             -0.0398500
      0.4607160              0.2696270
D    1   1.00
      0.2185020              1.0000000
D    1   1.00
      0.0987470              1.0000000
D    1   1.00
      0.0418720              1.0000000
F    3   1.00
      2.2991460              0.3237190
      1.0205490              0.3795530
      0.4433930              0.3340230
F    1   1.00
      0.1862010              1.0000000
F    1   1.00
      0.0715740              1.0000000
G    1   1.00
      0.5200000              1.0000000
G    1   1.00
      0.2200000              1.0000000
****

TH     0
TH-ECP     5     78
h potential
  1
2      1.000000000            0.000000000
s-h potential
  3
2      4.063653000          113.326466000
2      1.883995000           15.663755000
2      0.886567000           -2.765902000
p-h potential
  3
2      3.986181000          115.953483000
2      2.000160000           15.762190000
2      0.960841000            1.378850000
d-h potential
  3
2      4.147972000           59.747811000
2      2.234563000           17.820683000
2      0.913695000            6.913661000
f-h potential
  3
2      3.998938000           49.624378000
2      1.998840000          -26.641861000
2      0.995641000            1.762748000
g-h potential
  2
2      3.166379000          -28.595941000
2      0.860105000           -0.309653000


--Link1--
%OldChk=./3prime_pbe0.chk
%Chk=./3prime_lcwpbe.chk
# LC-wHPBE ChkBasis Guess(Read) Geom(Checkpoint) Int(SuperFineGrid) SCF(Symm,DSymm,FSymm,IntRep) Symmetry(PG=D3h)

3prime_lcwpbe

0 1


--Link1--
%OldChk=./3prime_lcwpbe.chk
%Chk=./3prime_dkhso.chk
# LC-wHPBE/Gen Guess(Read) Geom(Checkpoint) Int(SuperFineGrid,DKHSO) SCF(Symm,DSymm,FSymm,IntRep) Symmetry(PG=D3h) Pop(NBO7Read)

3prime_dkhso

0 1

H     0
S    3   1.00
     34.87758369             0.0233625
```

```
      5.23397281             0.1708758
      1.18503342             0.8057615
S    1   1.00
      0.32833943             1.0000000
S    1   1.00
      0.11031918             1.0000000
P    1   1.00
      0.71884400             1.0000000
P    1   1.00
      0.35000000             1.0000000
D    1   1.00
      1.07506000             1.0000000
****
C     0
S    5   1.00
   7615.05999110             0.0020534
   1145.08684615             0.0130533
    261.91952431             0.0643852
     74.66834196             0.2434904
     24.40551202             0.6770175
S    2   1.00
      8.68679013             0.5654885
      3.23657623             0.4345114
S    1   1.00
      1.30179999             1.0000000
S    1   1.00
      0.52768873             1.0000000
S    1   1.00
      0.15694534             1.0000000
P    4   1.00
     34.50767936             0.0105595
      7.91304441             0.0694749
      2.36615458             0.2809656
      0.81172425             0.6389997
P    1   1.00
      0.31228882             1.0000000
P    1   1.00
      0.11400266             1.0000000
D    1   1.00
      1.15404900             1.0000000
D    1   1.00
      0.35112860             1.0000000
F    1   1.00
      0.81931200             1.0000000
****
Cl     0
S    5   1.00
 181678.82698244             0.0042914
  27435.27709666             0.0160338
   6322.37997866             0.0586975
   1795.44303926             0.2141558
    575.07900250             0.7068212
S    2   1.00
      4.08968894            -0.6325784
      1.84597271            -0.3674215
S    1   1.00
    201.17812871             1.0000000
S    1   1.00
     74.61037542             1.0000000
S    1   1.00
     28.63265512             1.0000000
S    1   1.00
      8.23556833             1.0000000
S    1   1.00
      0.54888997             1.0000000
S    1   1.00
      0.19788882             1.0000000
P    6   1.00
    859.87676601             0.0016621
    218.96050941             0.0109967
     72.36853323             0.0519914
     26.91710141             0.1711570
     10.77497489             0.3550123
      4.52323960             0.4091802
P    1   1.00
      1.98716730             1.0000000
P    1   1.00
      0.90649824             1.0000000
P    1   1.00
      0.33862386             1.0000000
P    1   1.00
      0.11925000             1.0000000
D    1   1.00
      0.97515420             1.0000000
D    1   1.00
      0.32573949             1.0000000
F    1   1.00
      0.72996999             1.0000000
****
K     0
S    7   1.00
 428242.30424475             0.0008807
  65580.15994019             0.0028805
  15724.19413173             0.0088176
   4872.57135995             0.0257206
```

```
   1690.37044379              0.0803021
    598.80075308              0.2501246
    218.82354539              0.6312735
S    2   1.00
      4.63827453             -0.7740635
      2.08136315             -0.2259364
S    1   1.00
     84.45415215              1.0000000
S    1   1.00
     34.00275517             -1.0000000
S    1   1.00
     10.41692419              1.0000000
S    1   1.00
      0.74500339              1.0000000
S    1   1.00
      0.28931658             -1.0000000
S    1   1.00
      0.03508468              1.0000000
S    1   1.00
      0.01510794              1.0000000
P    5   1.00
   2570.25210920              0.0019666
    642.60993700              0.0119790
    222.45405690              0.0555607
     85.30416109              0.2243170
     34.01212437              0.7061766
P    3   1.00
      1.10953771              0.5278017
      0.45159540              0.3494904
      0.17668488              0.1227078
P    2   1.00
     14.08906645              0.4629581
      6.05154655              0.5370418
P    1   1.00
      2.67407506              1.0000000
P    1   1.00
      1.07698300              1.0000000
P    1   1.00
      0.03757100              1.0000000
D    1   1.00
      0.57805998              1.0000000
D    1   1.00
      0.09192000              1.0000000
F    1   1.00
      0.45136500              1.0000000
****
Th     0
S    8   1.00
16043244.65738062             0.0068383
2570568.37205177              0.0170456
 666162.47831555              0.0311353
 234516.27834475              0.0486144
  95166.94573264              0.0853333
  39869.48557288              0.1394299
  17299.31220432              0.2555934
   7368.51163727              0.4160093
S    3   1.00
   3062.32274268              0.5004687
   1297.71834244              0.3658576
    577.87190417              0.1336735
S    2   1.00
    238.44162611             -0.9552649
    113.11602561             -0.0447350
S    2   1.00
     42.85869717              0.9287246
     22.61188534              0.0712753
S    1   1.00
      8.73725993             -1.0000000
S    1   1.00
      1.81841519             -1.0000000
S    1   1.00
      0.46970981              1.0000000
S    1   1.00
      0.23200392              1.0000000
S    1   1.00
      0.11362006             -1.0000000
S    1   1.00
      0.05918614              1.0000000
P    7   1.00
  59561.94529738              0.0045472
  14733.92956117              0.0116735
   5264.43036673              0.0326093
   2254.59912122              0.0728472
    982.00937878              0.2044412
    426.29319086              0.3376818
    189.06940510              0.3361996
P    2   1.00
     79.06248291             -0.5150330
     37.90322417             -0.4849669
P    2   1.00
     16.07613158              0.5045826
      7.94210837              0.4954173
P    2   1.00
      3.13379048             -0.6247020
      1.52933840             -0.3752979
```

```
P    1   1.00
      0.57762182             1.0000000
P    1   1.00
      0.13584150             1.0000000
D    6   1.00
   3969.66987757             0.0023945
   1345.91282816             0.0127806
    552.61199007             0.0494451
    235.92901938             0.1909581
    104.96664922             0.3433713
     48.63266170             0.4010500
D    3   1.00
     22.04904780             0.4213352
     10.37206808             0.4032149
      4.73976567             0.1754497
D    1   1.00
      2.35121373             1.0000000
D    1   1.00
      1.17246913             1.0000000
D    1   1.00
      0.57941910             1.0000000
D    1   1.00
      0.27511954             1.0000000
F    4   1.00
    667.39099352             0.0056125
    225.98866045             0.0491476
     90.97449476             0.2432466
     39.59993497             0.7019932
F    1   1.00
     17.90506379             1.0000000
F    1   1.00
      7.14206950             1.0000000
F    1   1.00
      1.59064392             1.0000000
G    1   1.00
      5.43751929             1.0000000
G    1   1.00
      0.20779157             1.0000000
****

$NBO SKIPBO FIXDM BNDIDX FILE=3prime_dkhso DMNAO=W49 AONAO=W49 $END
```

3* (D_{3h}, *no imaginary frequencies*)

```
%Chk=3star_pbe0.chk
# PBE1PBE/GenECP EmpiricalDispersion=GD3BJ Int(SuperFineGrid) SCF(Symm,DSymm,FSymm,IntRep) Symmetry(PG=D3h,Follow,On)
Opt(RFO,Tight,CalcAll,NoEigenTest) Freq(Raman)

3star_pbe0

2 1
Th      -0.000000003825     -2.631857727161      0.000000000000
Cl      -1.746395616931     -1.008246188897      1.663554933774
C       -0.695377894992     -4.556112735077      1.699499940863
H       -1.106740834434     -4.467394383673      2.700422919888
Th       2.279255651431      1.315928857932      0.000000000000
Cl       1.746364619971     -1.008299877207      1.663554933774
C       -1.692175486175     -4.557416790831      0.711158340180
H       -2.689096880368     -4.455934799094      1.128457266244
Cl       1.746395614001     -1.008246193973     -1.663554933774
C       -1.698989292888     -4.557371420369     -0.694671001122
H       -2.699919476997     -4.455879674294     -1.102283764725
C       -0.711885201477     -4.556172801670     -1.692671527793
H       -1.132962943734     -4.467183249307     -2.689506562770
C        0.695377881750     -4.556112737098     -1.699499940863
H        1.106740821449     -4.467394386889     -2.700422919888
C        1.692175472929     -4.557416795749     -0.711158340180
H        2.689096867417     -4.455934806910     -1.128457266244
C        1.698989279642     -4.557371425307      0.694671001122
H        2.699919464046     -4.455879682141      1.102283764725
C        0.711885188234     -4.556172803739      1.692671527793
H        1.132962930750     -4.467183252600      2.689506562770
C        3.598020430609      2.880271277038     -1.699499940863
H        3.315506615697      3.192162857889     -2.700422919888
C        4.792926458233      0.813241434391      0.711158340180
H        5.203501172458     -0.100858814425      1.128457266244
C        4.796294069616      0.807317819450     -0.694671001122
H        5.208864731295     -0.110259020441     -1.102283764725
C        4.301703989668      1.661575729443     -1.692671527793
H        4.435175647779      1.252416931498     -2.689506562770
C        4.293398317230      1.675841442910      1.699499940863
H        4.422247440853      1.275231511475      2.700422919888
C        3.100750982940      3.744175342757     -0.711158340180
H        2.514404305334      4.556793601539     -1.128457266244
C        3.097304787628      3.750053587245      0.694671001122
H        2.508945267570      4.566138682771      1.102283764725
C        3.589818796604      2.894597057123      1.692671527793
H        3.302212713389      3.214766303540      2.689506562770
Th      -2.279255647607      1.315928864557      0.000000000000
```

```
Cl       -1.746364622902     -1.008299872131     -1.663554933774
C        -4.293398312359      1.675841455388     -1.699499940863
H        -4.422247437146      1.275231524329     -2.700422919888
Cl       -0.000030991099      2.016546061433     -1.663554933774
C        -4.792926455869      0.813241448322     -0.711158340180
H        -5.203501172751     -0.100858799301     -1.128457266244
Cl        0.000030996960      2.016546061433      1.663554933774
C        -4.796294067270      0.807317833391      0.694671001122
H        -5.208864731616     -0.110259005301      1.102283764725
C        -4.301703984839      1.661575741945      1.692671527793
H        -4.435175644139      1.252416944389      2.689506562770
C        -3.598020422238      2.880271287496      1.699499940863
H        -3.315506606419      3.192162867526      2.700422919888
C        -3.100750972057      3.744175351769      0.711158340180
H        -2.514404292090      4.556793608847      1.128457266244
C        -3.097304776728      3.750053596247     -0.694671001122
H        -2.508945254298      4.566138690063     -1.102283764725
C        -3.589818788191      2.894597067557     -1.692671527793
H        -3.302212704045      3.214766313138     -2.689506562770
K         0.000000000000     -0.000000001557     -4.049388009927
K         0.000000000000     -0.000000001557      4.049388009927

C Cl 0
cc-pVTZ
****
H 0
cc-pVDZ
****
K 0
6-311G*
****
Th     0
S    4   1.00
     10.5166570             0.0330960
      7.0109060            -0.2339080
      4.6738090             0.5198480
      1.8620810            -1.0189420
S    1   1.00
      0.5373270             1.0000000
S    1   1.00
      0.2614820             1.0000000
S    1   1.00
      0.0682570             1.0000000
S    1   1.00
      0.0275220             1.0000000
P    4   1.00
     15.8728110             0.0015590
     10.5818740            -0.0069170
      3.9249540             0.1150440
      2.2082920            -0.3349900
P    1   1.00
      0.4929470             1.0000000
P    1   1.00
      0.2151610             1.0000000
P    1   1.00
      0.0545080             1.0000000
P    1   1.00
      0.0143650             1.0000000
D    3   1.00
      6.0714120             0.0017310
      1.9840490            -0.0398500
      0.4607160             0.2696270
D    1   1.00
      0.2185020             1.0000000
D    1   1.00
      0.0987470             1.0000000
D    1   1.00
      0.0418720             1.0000000
F    3   1.00
      2.2991460             0.3237190
      1.0205490             0.3795530
      0.4433930             0.3340230
F    1   1.00
      0.1862010             1.0000000
F    1   1.00
      0.0715740             1.0000000
G    1   1.00
      0.5200000             1.0000000
G    1   1.00
      0.2200000             1.0000000
****

TH     0
TH-ECP     5     78
h potential
  1
2      1.000000000            0.000000000
s-h potential
  3
2      4.063653000          113.326466000
2      1.883995000           15.663755000
2      0.886567000           -2.765902000
p-h potential
  3
2      3.986181000          115.953483000
```

```
2      2.000160000           15.762190000
2      0.960841000            1.378850000
d-h potential
  3
2      4.147972000           59.747811000
2      2.234563000           17.820683000
2      0.913695000            6.913661000
f-h potential
  3
2      3.998938000           49.624378000
2      1.998840000          -26.641861000
2      0.995641000            1.762748000
g-h potential
  2
2      3.166379000          -28.595941000
2      0.860105000           -0.309653000


--Link1--
%OldChk=./3star_pbe0.chk
%Chk=./3star_lcwpbe.chk
# LC-wHPBE ChkBasis Guess(Read) Geom(Checkpoint) Int(SuperFineGrid) SCF(Symm,DSymm,FSymm,IntRep) Symmetry(PG=D3h)

3star_lcwpbe

2 1


--Link1--
%OldChk=./3star_lcwpbe.chk
%Chk=./3star_dkhso.chk
# LC-wHPBE/Gen Guess(Read) Geom(Checkpoint) Int(SuperFineGrid,DKHSO) SCF(Symm,DSymm,FSymm,IntRep) Symmetry(PG=D3h) Pop(NBO7Read)

3star_dkhso

2 1

H     0
S    3   1.00
     34.87758369             0.0233625
      5.23397281             0.1708758
      1.18503342             0.8057615
S    1   1.00
      0.32833943             1.0000000
S    1   1.00
      0.11031918             1.0000000
P    1   1.00
      0.71884400             1.0000000
P    1   1.00
      0.35000000             1.0000000
D    1   1.00
      1.07506000             1.0000000
****
C     0
S    5   1.00
   7615.05999110             0.0020534
   1145.08684615             0.0130533
    261.91952431             0.0643852
     74.66834196             0.2434904
     24.40551202             0.6770175
S    2   1.00
      8.68679013             0.5654885
      3.23657623             0.4345114
S    1   1.00
      1.30179999             1.0000000
S    1   1.00
      0.52768873             1.0000000
S    1   1.00
      0.15694534             1.0000000
P    4   1.00
     34.50767936             0.0105595
      7.91304441             0.0694749
      2.36615458             0.2809656
      0.81172425             0.6389997
P    1   1.00
      0.31228882             1.0000000
P    1   1.00
      0.11400266             1.0000000
D    1   1.00
      1.15404900             1.0000000
D    1   1.00
      0.35112860             1.0000000
F    1   1.00
      0.81931200             1.0000000
****
Cl     0
S    5   1.00
 181678.82698244             0.0042914
  27435.27709666             0.0160338
   6322.37997866             0.0586975
   1795.44303926             0.2141558
    575.07900250             0.7068212
S    2   1.00
      4.08968894            -0.6325784
      1.84597271            -0.3674215
```

```
S    1   1.00
    201.17812871             1.0000000
S    1   1.00
     74.61037542             1.0000000
S    1   1.00
     28.63265512             1.0000000
S    1   1.00
      8.23556833             1.0000000
S    1   1.00
      0.54888997             1.0000000
S    1   1.00
      0.19788882             1.0000000
P    6   1.00
    859.87676601             0.0016621
    218.96050941             0.0109967
     72.36853323             0.0519914
     26.91710141             0.1711570
     10.77497489             0.3550123
      4.52323960             0.4091802
P    1   1.00
      1.98716730             1.0000000
P    1   1.00
      0.90649824             1.0000000
P    1   1.00
      0.33862386             1.0000000
P    1   1.00
      0.11925000             1.0000000
D    1   1.00
      0.97515420             1.0000000
D    1   1.00
      0.32573949             1.0000000
F    1   1.00
      0.72996999             1.0000000
****
K     0
S    7   1.00
 428242.30424475             0.0008807
  65580.15994019             0.0028805
  15724.19413173             0.0088176
   4872.57135995             0.0257206
   1690.37044379             0.0803021
    598.80075308             0.2501246
    218.82354539             0.6312735
S    2   1.00
      4.63827453            -0.7740635
      2.08136315            -0.2259364
S    1   1.00
     84.45415215             1.0000000
S    1   1.00
     34.00275517            -1.0000000
S    1   1.00
     10.41692419             1.0000000
S    1   1.00
      0.74500339             1.0000000
S    1   1.00
      0.28931658            -1.0000000
S    1   1.00
      0.03508468             1.0000000
S    1   1.00
      0.01510794             1.0000000
P    5   1.00
   2570.25210920             0.0019666
    642.60993700             0.0119790
    222.45405690             0.0555607
     85.30416109             0.2243170
     34.01212437             0.7061766
P    3   1.00
      1.10953771             0.5278017
      0.45159540             0.3494904
      0.17668488             0.1227078
P    2   1.00
     14.08906645             0.4629581
      6.05154655             0.5370418
P    1   1.00
      2.67407506             1.0000000
P    1   1.00
      1.07698300             1.0000000
P    1   1.00
      0.03757100             1.0000000
D    1   1.00
      0.57805998             1.0000000
D    1   1.00
      0.09192000             1.0000000
F    1   1.00
      0.45136500             1.0000000
****
Th     0
S    8   1.00
16043244.65738062            0.0068383
2570568.37205177             0.0170456
 666162.47831555             0.0311353
 234516.27834475             0.0486144
  95166.94573264             0.0853333
  39869.48557288             0.1394299
  17299.31220432             0.2555934
```

```
   7368.51163727             0.4160093
S    3   1.00
   3062.32274268             0.5004687
   1297.71834244             0.3658576
    577.87190417             0.1336735
S    2   1.00
    238.44162611            -0.9552649
    113.11602561            -0.0447350
S    2   1.00
     42.85869717             0.9287246
     22.61188534             0.0712753
S    1   1.00
      8.73725993            -1.0000000
S    1   1.00
      1.81841519            -1.0000000
S    1   1.00
      0.46970981             1.0000000
S    1   1.00
      0.23200392             1.0000000
S    1   1.00
      0.11362006            -1.0000000
S    1   1.00
      0.05918614             1.0000000
P    7   1.00
  59561.94529738             0.0045472
  14733.92956117             0.0116735
   5264.43036673             0.0326093
   2254.59912122             0.0728472
    982.00937878             0.2044412
    426.29319086             0.3376818
    189.06940510             0.3361996
P    2   1.00
     79.06248291            -0.5150330
     37.90322417            -0.4849669
P    2   1.00
     16.07613158             0.5045826
      7.94210837             0.4954173
P    2   1.00
      3.13379048            -0.6247020
      1.52933840            -0.3752979
P    1   1.00
      0.57762182             1.0000000
P    1   1.00
      0.13584150             1.0000000
D    6   1.00
   3969.66987757             0.0023945
   1345.91282816             0.0127806
    552.61199007             0.0494451
    235.92901938             0.1909581
    104.96664922             0.3433713
     48.63266170             0.4010500
D    3   1.00
     22.04904780             0.4213352
     10.37206808             0.4032149
      4.73976567             0.1754497
D    1   1.00
      2.35121373             1.0000000
D    1   1.00
      1.17246913             1.0000000
D    1   1.00
      0.57941910             1.0000000
D    1   1.00
      0.27511954             1.0000000
F    4   1.00
    667.39099352             0.0056125
    225.98866045             0.0491476
     90.97449476             0.2432466
     39.59993497             0.7019932
F    1   1.00
     17.90506379             1.0000000
F    1   1.00
      7.14206950             1.0000000
F    1   1.00
      1.59064392             1.0000000
G    1   1.00
      5.43751929             1.0000000
G    1   1.00
      0.20779157             1.0000000
****

$NBO SKIPBO FIXDM BNDIDX FILE=3star_dkhso DMNAO=W49 AONAO=W49 $END
```

3† **(** D$_{3h}$, *no imaginary frequencies*)

```
%Chk=3dagger_pbe0.chk
# PBE1PBE/GenECP EmpiricalDispersion=GD3BJ Int(SuperFineGrid) SCF(Symm,DSymm,FSymm,IntRep) Symmetry(PG=D3h,Follow,On)
Opt(RFO,Tight,CalcAll,NoEigenTest) Freq(Raman)

3dagger_pbe0

0 1
Th       -2.197569238553      1.268767199687      0.000000000000
Cl        0.000000004572      2.004361352945      1.647008713912
C        -3.548904104412      2.860658519291      1.696062479293
H        -3.235338681306      3.157108502700      2.692307910386
Th       -0.000000005788     -2.537534384662      0.000000000000
Cl       -1.735827850947     -1.002180670170      1.647008713912
C        -3.058336904652      3.721788079373      0.702709473970
H        -2.454255140604      4.524148843213      1.114701047358
Cl       -1.735827850947     -1.002180670170     -1.647008713912
C        -3.058336904652      3.721788079373     -0.702709473970
H        -2.454255140604      4.524148843213     -1.114701047358
C        -3.548904104412      2.860658519291     -1.696062479293
H        -3.235338681306      3.157108502700     -2.692307910386
C        -4.251854992615      1.643111872111     -1.696062479293
H        -4.351805499561      1.223331258704     -2.692307910386
C        -4.752331471618      0.787703437097     -0.702709473970
H        -5.145155398296     -0.136627096658     -1.114701047358
C        -4.752331471618      0.787703437097      0.702709473970
H        -5.145155398296     -0.136627096658      1.114701047358
C        -4.251854992615      1.643111872111      1.696062479293
H        -4.351805499561      1.223331258704      2.692307910386
C         0.702950875153     -4.503770370525     -1.696062479293
H         1.116466803852     -4.380439741957     -2.692307910386
C        -1.693994570560     -4.509491490102      0.702709473970
H        -2.690900257069     -4.387521718394      1.114701047358
C        -1.693994570560     -4.509491490102     -0.702709473970
H        -2.690900257069     -4.387521718394     -1.114701047358
C        -0.702950895699     -4.503770367318     -1.696062479293
H        -1.116466823836     -4.380439736864     -2.692307910386
C        -0.702950895699     -4.503770367318      1.696062479293
H        -1.116466823836     -4.380439736864      2.692307910386
C         1.693994549987     -4.509491497830     -0.702709473970
H         2.690900237053     -4.387521730670     -1.114701047358
C         1.693994549987     -4.509491497830      0.702709473970
H         2.690900237053     -4.387521730670      1.114701047358
C         0.702950875153     -4.503770370525      1.696062479293
H         1.116466803852     -4.380439741957      2.692307910386
Th        2.197569244341      1.268767189662      0.000000000000
Cl        0.000000004572      2.004361352945     -1.647008713912
C         3.548904117463      2.860658503101     -1.696062479293
H         3.235338695709      3.157108487940     -2.692307910386
Cl        1.735827846375     -1.002180678088     -1.647008713912
C         3.058336921631      3.721788065421     -0.702709473970
H         2.454255161243      4.524148832016     -1.114701047358
Cl        1.735827846375     -1.002180678088      1.647008713912
C         3.058336921631      3.721788065421      0.702709473970
H         2.454255161243      4.524148832016      1.114701047358
C         3.548904117463      2.860658503101      1.696062479293
H         3.235338695709      3.157108487940      2.692307910386
C         4.251855000111      1.643111852714      1.696062479293
H         4.351805505142      1.223331238851      2.692307910386
C         4.752331475212      0.787703415417      0.702709473970
H         5.145155397673     -0.136627120131      1.114701047358
C         4.752331475212      0.787703415417     -0.702709473970
H         5.145155397673     -0.136627120131     -1.114701047358
C         4.251855000111      1.643111852714     -1.696062479293
H         4.351805505142      1.223331238851     -2.692307910386
Ar        0.000000000000      0.000000001562     -4.868617302798
Ar        0.000000000000      0.000000001562      4.868617302798

C Cl 0
cc-pVTZ
****
H 0
cc-pVDZ
****
Ar 0
6-311G*
****
Th     0
S    4   1.00
     10.5166570             0.0330960
      7.0109060            -0.2339080
      4.6738090             0.5198480
      1.8620810            -1.0189420
S    1   1.00
      0.5373270             1.0000000
S    1   1.00
      0.2614820             1.0000000
S    1   1.00
      0.0682570             1.0000000
S    1   1.00
```

```
      0.0275220              1.0000000
P    4   1.00
     15.8728110              0.0015590
     10.5818740             -0.0069170
      3.9249540              0.1150440
      2.2082920             -0.3349900
P    1   1.00
      0.4929470              1.0000000
P    1   1.00
      0.2151610              1.0000000
P    1   1.00
      0.0545080              1.0000000
P    1   1.00
      0.0143650              1.0000000
D    3   1.00
      6.0714120              0.0017310
      1.9840490             -0.0398500
      0.4607160              0.2696270
D    1   1.00
      0.2185020              1.0000000
D    1   1.00
      0.0987470              1.0000000
D    1   1.00
      0.0418720              1.0000000
F    3   1.00
      2.2991460              0.3237190
      1.0205490              0.3795530
      0.4433930              0.3340230
F    1   1.00
      0.1862010              1.0000000
F    1   1.00
      0.0715740              1.0000000
G    1   1.00
      0.5200000              1.0000000
G    1   1.00
      0.2200000              1.0000000
****

TH     0
TH-ECP     5     78
h potential
  1
2      1.000000000            0.000000000
s-h potential
  3
2      4.063653000          113.326466000
2      1.883995000           15.663755000
2      0.886567000           -2.765902000
p-h potential
  3
2      3.986181000          115.953483000
2      2.000160000           15.762190000
2      0.960841000            1.378850000
d-h potential
  3
2      4.147972000           59.747811000
2      2.234563000           17.820683000
2      0.913695000            6.913661000
f-h potential
  3
2      3.998938000           49.624378000
2      1.998840000          -26.641861000
2      0.995641000            1.762748000
g-h potential
  2
2      3.166379000          -28.595941000
2      0.860105000           -0.309653000


--Link1--
%OldChk=./3dagger_pbe0.chk
%Chk=./3dagger_lcwpbe.chk
# LC-wHPBE ChkBasis Guess(Read) Geom(Checkpoint) Int(SuperFineGrid) SCF(Symm,DSymm,FSymm,IntRep) Symmetry(PG=D3h)

3dagger_lcwpbe

0 1


--Link1--
%OldChk=./3dagger_lcwpbe.chk
%Chk=./3dagger_dkhso.chk
# LC-wHPBE/Gen Guess(Read) Geom(Checkpoint) Int(SuperFineGrid,DKHSO) SCF(Symm,DSymm,FSymm,IntRep) Symmetry(PG=D3h) Pop(NBO7Read)

3dagger_dkhso

0 1

H     0
S    3   1.00
     34.87758369             0.0233625
      5.23397281             0.1708758
      1.18503342             0.8057615
S    1   1.00
      0.32833943             1.0000000
```

```
S    1   1.00
      0.11031918             1.0000000
P    1   1.00
      0.71884400             1.0000000
P    1   1.00
      0.35000000             1.0000000
D    1   1.00
      1.07506000             1.0000000
****
C     0
S    5   1.00
   7615.05999110             0.0020534
   1145.08684615             0.0130533
    261.91952431             0.0643852
     74.66834196             0.2434904
     24.40551202             0.6770175
S    2   1.00
      8.68679013             0.5654885
      3.23657623             0.4345114
S    1   1.00
      1.30179999             1.0000000
S    1   1.00
      0.52768873             1.0000000
S    1   1.00
      0.15694534             1.0000000
P    4   1.00
     34.50767936             0.0105595
      7.91304441             0.0694749
      2.36615458             0.2809656
      0.81172425             0.6389997
P    1   1.00
      0.31228882             1.0000000
P    1   1.00
      0.11400266             1.0000000
D    1   1.00
      1.15404900             1.0000000
D    1   1.00
      0.35112860             1.0000000
F    1   1.00
      0.81931200             1.0000000
****
Cl     0
S    5   1.00
 181678.82698244             0.0042914
  27435.27709666             0.0160338
   6322.37997866             0.0586975
   1795.44303926             0.2141558
    575.07900250             0.7068212
S    2   1.00
      4.08968894            -0.6325784
      1.84597271            -0.3674215
S    1   1.00
    201.17812871             1.0000000
S    1   1.00
     74.61037542             1.0000000
S    1   1.00
     28.63265512             1.0000000
S    1   1.00
      8.23556833             1.0000000
S    1   1.00
      0.54888997             1.0000000
S    1   1.00
      0.19788882             1.0000000
P    6   1.00
    859.87676601             0.0016621
    218.96050941             0.0109967
     72.36853323             0.0519914
     26.91710141             0.1711570
     10.77497489             0.3550123
      4.52323960             0.4091802
P    1   1.00
      1.98716730             1.0000000
P    1   1.00
      0.90649824             1.0000000
P    1   1.00
      0.33862386             1.0000000
P    1   1.00
      0.11925000             1.0000000
D    1   1.00
      0.97515420             1.0000000
D    1   1.00
      0.32573949             1.0000000
F    1   1.00
      0.72996999             1.0000000
****
Ar     0
S    5   1.00
 163430.88636138             0.0055979
  24462.98316426             0.0210633
   5847.39155450             0.0713189
   1838.33954786             0.2210048
    646.47797239             0.6810149
S    2   1.00
    233.37419769             0.3557513
     87.35339236             0.6442486
```

```
S    1   1.00
     35.25747909             1.0000000
S    1   1.00
     14.90945611             1.0000000
S    1   1.00
      5.16577808             1.0000000
S    1   1.00
      2.13251221             1.0000000
S    1   1.00
      0.66446873             1.0000000
S    1   1.00
      0.23088302             1.0000000
P    6   1.00
   1434.65644528             0.0010121
    344.67516841             0.0071471
    111.24392588             0.0365960
     39.84545454             0.1410979
     15.22684292             0.3487092
      6.12548056             0.4654374
P    1   1.00
      2.61266778             1.0000000
P    1   1.00
      1.09059943             1.0000000
P    1   1.00
      0.40193082             1.0000000
P    1   1.00
      0.14113163             1.0000000
D    1   1.00
      1.19109400             1.0000000
D    1   1.00
      0.39315500             1.0000000
F    1   1.00
      0.90998994             1.0000000
****
Th     0
S    8   1.00
16043244.65738062            0.0068383
2570568.37205177             0.0170456
 666162.47831555             0.0311353
 234516.27834475             0.0486144
  95166.94573264             0.0853333
  39869.48557288             0.1394299
  17299.31220432             0.2555934
   7368.51163727             0.4160093
S    3   1.00
   3062.32274268             0.5004687
   1297.71834244             0.3658576
    577.87190417             0.1336735
S    2   1.00
    238.44162611            -0.9552649
    113.11602561            -0.0447350
S    2   1.00
     42.85869717             0.9287246
     22.61188534             0.0712753
S    1   1.00
      8.73725993            -1.0000000
S    1   1.00
      1.81841519            -1.0000000
S    1   1.00
      0.46970981             1.0000000
S    1   1.00
      0.23200392             1.0000000
S    1   1.00
      0.11362006            -1.0000000
S    1   1.00
      0.05918614             1.0000000
P    7   1.00
  59561.94529738             0.0045472
  14733.92956117             0.0116735
   5264.43036673             0.0326093
   2254.59912122             0.0728472
    982.00937878             0.2044412
    426.29319086             0.3376818
    189.06940510             0.3361996
P    2   1.00
     79.06248291            -0.5150330
     37.90322417            -0.4849669
P    2   1.00
     16.07613158             0.5045826
      7.94210837             0.4954173
P    2   1.00
      3.13379048            -0.6247020
      1.52933840            -0.3752979
P    1   1.00
      0.57762182             1.0000000
P    1   1.00
      0.13584150             1.0000000
D    6   1.00
   3969.66987757             0.0023945
   1345.91282816             0.0127806
    552.61199007             0.0494451
    235.92901938             0.1909581
    104.96664922             0.3433713
     48.63266170             0.4010500
D    3   1.00
```

```
     22.04904780             0.4213352
     10.37206808             0.4032149
      4.73976567             0.1754497
D    1   1.00
      2.35121373             1.0000000
D    1   1.00
      1.17246913             1.0000000
D    1   1.00
      0.57941910             1.0000000
D    1   1.00
      0.27511954             1.0000000
F    4   1.00
    667.39099352             0.0056125
    225.98866045             0.0491476
     90.97449476             0.2432466
     39.59993497             0.7019932
F    1   1.00
     17.90506379             1.0000000
F    1   1.00
      7.14206950             1.0000000
F    1   1.00
      1.59064392             1.0000000
G    1   1.00
      5.43751929             1.0000000
G    1   1.00
      0.20779157             1.0000000
****

$NBO SKIPBO FIXDM BNDIDX FILE=3dagger_dkhso DMNAO=W49 AONAO=W49 $END
```

COT^{2+} (D_{8h}, *planarized, 2 imaginary frequencies*)

```
%Chk=COT6pi_pbe0.chk
# PBE1PBE/Gen EmpiricalDispersion=GD3BJ Int(SuperFineGrid) SCF(Symm,DSymm,FSymm,IntRep) Symmetry(PG=D8h,Follow,On)
Opt(RFO,Tight,CalcAll,NoEigenTest) Freq

COT6pi_pbe0

2 1
C                 -0.700263    1.690586    0.000000
C                 -1.690586    0.700263    0.000000
C                 -1.690586   -0.700263    0.000000
C                 -0.700263   -1.690586    0.000000
C                  0.700263   -1.690586    0.000000
C                  1.690586   -0.700263    0.000000
C                  1.690586    0.700263    0.000000
C                  0.700263    1.690586    0.000000
H                 -1.117605    2.698133    0.000000
H                 -2.698133    1.117605    0.000000
H                 -2.698133   -1.117605    0.000000
H                 -1.117605   -2.698133    0.000000
H                  1.117605   -2.698133    0.000000
H                  2.698133   -1.117605    0.000000
H                  2.698133    1.117605    0.000000
H                  1.117605    2.698133    0.000000

C 0
cc-pVTZ
****
H 0
cc-pVDZ
****


--Link1--
%OldChk=./COT6pi_pbe0.chk
%Chk=./COT6pi_lcwpbe.chk
# LC-wHPBE ChkBasis Guess(Read) Geom(Checkpoint) Int(SuperFineGrid) SCF(Symm,DSymm,FSymm,IntRep) Symmetry(PG=D8h)

COT6pi_lcwpbe

2 1


--Link1--
%OldChk=./COT6pi_lcwpbe.chk
%Chk=./COT6pi_dkhso.chk
# LC-wHPBE/Gen Guess(Read) Geom(Checkpoint) Int(SuperFineGrid,DKHSO) SCF(Symm,DSymm,FSymm,IntRep) Symmetry(PG=D8h) Pop(NBO7Read)

COT6pi_dkhso

2 1

H     0
S    3   1.00
     34.87758369             0.0233625
      5.23397281             0.1708758
```

```
     1.18503342             0.8057615
S    1   1.00
     0.32833943             1.0000000
S    1   1.00
     0.11031918             1.0000000
P    1   1.00
     0.71884400             1.0000000
P    1   1.00
     0.35000000             1.0000000
D    1   1.00
     1.07506000             1.0000000
****
C     0
S    5   1.00
  7615.05999110             0.0020534
  1145.08684615             0.0130533
   261.91952431             0.0643852
    74.66834196             0.2434904
    24.40551202             0.6770175
S    2   1.00
     8.68679013             0.5654885
     3.23657623             0.4345114
S    1   1.00
     1.30179999             1.0000000
S    1   1.00
     0.52768873             1.0000000
S    1   1.00
     0.15694534             1.0000000
P    4   1.00
    34.50767936             0.0105595
     7.91304441             0.0694749
     2.36615458             0.2809656
     0.81172425             0.6389997
P    1   1.00
     0.31228882             1.0000000
P    1   1.00
     0.11400266             1.0000000
D    1   1.00
     1.15404900             1.0000000
D    1   1.00
     0.35112860             1.0000000
F    1   1.00
     0.81931200             1.0000000
****

$NBO SKIPBO FIXDM BNDIDX FILE=COT6pi_dkhso DMNAO=W49 AONAO=W49 $END
```

COT^{2-} (D$_{8h}$, *no imaginary frequencies*)

```
%Chk=COT10pi_pbe0.chk
# PBE1PBE/Gen EmpiricalDispersion=GD3BJ Int(SuperFineGrid) SCF(Symm,DSymm,FSymm,IntRep) Symmetry(PG=D8h,Follow,On) Opt(RFO,Tight)
Freq

COT10pi_pbe0

-2 1
C                 -0.704228    1.700159    0.000000
C                 -1.700159    0.704228    0.000000
C                 -1.700159   -0.704228    0.000000
C                 -0.704228   -1.700159    0.000000
C                  0.704228   -1.700159    0.000000
C                  1.700159   -0.704228    0.000000
C                  1.700159    0.704228    0.000000
C                  0.704228    1.700159    0.000000
H                 -1.124758    2.715407    0.000000
H                 -2.715407    1.124758    0.000000
H                 -2.715407   -1.124758    0.000000
H                 -1.124758   -2.715407    0.000000
H                  1.124758   -2.715407    0.000000
H                  2.715407   -1.124758    0.000000
H                  2.715407    1.124758    0.000000
H                  1.124758    2.715407    0.000000

C 0
cc-pVTZ
****
H 0
cc-pVDZ
****


--Link1--
%OldChk=./COT10pi_pbe0.chk
%Chk=./COT10pi_lcwpbe.chk
# LC-wHPBE ChkBasis Guess(Read) Geom(Checkpoint) Int(SuperFineGrid) SCF(Symm,DSymm,FSymm,IntRep) Symmetry(PG=D8h)

COT10pi_lcwpbe
```

```
-2 1


--Link1--
%OldChk=./COT10pi_lcwpbe.chk
%Chk=./COT10pi_dkhso.chk
# LC-wHPBE/Gen Guess(Read) Geom(Checkpoint) Int(SuperFineGrid,DKHSO) SCF(Symm,DSymm,FSymm,IntRep) Symmetry(PG=D8h) Pop(NBO7Read)

COT10pi_dkhso

-2 1

H     0
S    3   1.00
     34.87758369             0.0233625
      5.23397281             0.1708758
      1.18503342             0.8057615
S    1   1.00
      0.32833943             1.0000000
S    1   1.00
      0.11031918             1.0000000
P    1   1.00
      0.71884400             1.0000000
P    1   1.00
      0.35000000             1.0000000
D    1   1.00
      1.07506000             1.0000000
****
C     0
S    5   1.00
   7615.05999110             0.0020534
   1145.08684615             0.0130533
    261.91952431             0.0643852
     74.66834196             0.2434904
     24.40551202             0.6770175
S    2   1.00
      8.68679013             0.5654885
      3.23657623             0.4345114
S    1   1.00
      1.30179999             1.0000000
S    1   1.00
      0.52768873             1.0000000
S    1   1.00
      0.15694534             1.0000000
P    4   1.00
     34.50767936             0.0105595
      7.91304441             0.0694749
      2.36615458             0.2809656
      0.81172425             0.6389997
P    1   1.00
      0.31228882             1.0000000
P    1   1.00
      0.11400266             1.0000000
D    1   1.00
      1.15404900             1.0000000
D    1   1.00
      0.35112860             1.0000000
F    1   1.00
      0.81931200             1.0000000
****

$NBO SKIPBO FIXDM BNDIDX FILE=COT10pi_dkhso DMNAO=W49 AONAO=W49 $END
```

References

1. Gaussian 16, Revision C.01, Frisch, M. J.; Trucks, G. W.; Schlegel, H. B.; Scuseria, G. E.; Robb, M. A.; Cheeseman, J. R.; Scalmani, G.; Barone, V.; Petersson, G. A.; Nakatsuji, H.; Li, X.; Caricato, M.; Marenich, A. V.; Bloino, J.; Janesko, B. G.; Gomperts, R.; Mennucci, B.; Hratchian, H. P.; Ortiz, J. V.; Izmaylov, A. F.; Sonnenberg, J. L.; Williams-Young, D.; Ding, F.; Lipparini, F.; Egidi, F.; Goings, J.; Peng, B.; Petrone, A.; Henderson, T.; Ranasinghe, D.; Zakrzewski, V. G.; Gao, J.; Rega, N.; Zheng, G.; Liang, W.; Hada, M.; Ehara, M.; Toyota, K.; Fukuda, R.; Hasegawa, J.; Ishida, M.; Nakajima, T.; Honda, Y.; Kitao, O.; Nakai, H.; Vreven, T.; Throssell, K.; Montgomery, J. A., Jr.; Peralta, J. E.; Ogliaro, F.; Bearpark, M. J.; Heyd, J. J.; Brothers, E. N.; Kudin, K. N.; Staroverov, V. N.; Keith, T. A.; Kobayashi, R.; Normand, J.; Raghavachari, K.; Rendell, A. P.; Burant, J. C.; Iyengar, S. S.; Tomasi, J.; Cossi, M.; Millam, J. M.; Klene, M.; Adamo, C.; Cammi, R.; Ochterski, J. W.; Martin, R. L.; Morokuma, K.; Farkas, O.; Foresman, J. B.; Fox, D. J. Gaussian, Inc., Wallingford CT, 2016.

2. Boronski, J.T., Seed, J.A., Hunger, D., Woodward A.W., van Slageren J., Wooles A.J., L.S. Natrajan, Kaltsoyannis N., Liddle S.T. A crystalline tri-thorium cluster with σ-aromatic metal–metal bonding. *Nature* **598**, 72–75 (2021).

3. Adamo, C., Barone, V. Toward reliable density functional methods without adjustable parameters: The PBE0 model. *J. Chem. Phys.* **110**, 6158–6170 (1999).

4. Küchle, W., Dolg, M., Stoll, H., Preuss, H. J. Energy-adjusted pseudopotentials for the actinides. Parameter sets and test calculations for thorium and thorium monoxide. *J. Chem. Phys.* **100**, 7535–7542 (1994).

5. Cao, X., Dolg, M. Segmented contraction scheme for small-core actinide pseudopotential basis sets. *J. Mol. Struct. THEOCHEM* **673**, 203–209 (2004).

6. Cao, X., Dolg, M., Stoll, H. J. Valence basis sets for relativistic energy-consistent small-core actinide pseudopotentials. *J. Chem. Phys.* **118**, 487–496 (2003).

7. Grimme, S., Antony, J., Ehrlich, S., Krieg, H. A. A consistent and accurate ab initio parametrization of density functional dispersion correction (DFT-D) for the 94 elements H-Pu. *J. Chem. Phys.* **132**, 154104 (2010).

8. Becke, A. D., Johnson, E. R. A density-functional model of the dispersion interaction. *J. Chem. Phys.* **123**, 154101 (2005).

9. Johnson, E. R., Becke, A. D. A post-Hartree–Fock model of intermolecular interactions. *J. Chem. Phys.* **123**, 024101 (2005).

10. Johnson, E. R., Becke, A. D. A post-Hartree-Fock model of intermolecular interactions: Inclusion of higher-order corrections. *J. Chem. Phys.* **124**, 174104 (2006).

11. Simons, J., Jørgensen, P., Taylor, H., Ozment, J. Walking on Potential Energy Surfaces. *J. Phys. Chem.* **87**, 2745-53 (1983).

12. Szczepanik, D.W., Solà, M., Andrzejak, M., Pawełek, B., Dominikowska, J., Kukułka, M., Dyduch, K., Krygowski, T.M., Szatylowicz, H. The role of the long-range exchange corrections in the description of electron delocalization in aromatic species. *J. Comput. Chem.* **38**, 1640−1654 (2017).

13. Henderson, T. M., Izmaylov, A. F., Scalmani, G., Scuseria, G. E. Can short-range hybrids describe long-range-dependent properties? *J. Chem. Phys.* **131**, 044108 (2009).

14. Douglas, M., Kroll, N.M. Quantum electrodynamical corrections to the fine structure of helium. *Ann. Phys.* **82**, 89–155 (1974).

15. Hess, B.A. Applicability of the no-pair equation with free-particle projection operators to atomic and molecular structure calculations. *Phys. Rev. A* **32**, 756–763 (1985).

16. Hess, B.A. Relativistic electronic-structure calculations employing a two-component no-pair formalism with external-field projection operators. *Phys. Rev. A* **33**, 3742–3748 (1986).

17. de Oliveira, A. Z., Campos, C. T., Jorge, F. E., Ferreira, I. B., Fantin, P. A. All-electron triple zeta basis sets for the actinides. *Comput. Theor. Chem.* **1135**, 28–33 (2018).

18. Szczepanik, D.W., Solà, M. The electron density of delocalized bonds (EDDB) as a measure of local and global aromaticity. "Aromaticity: Modern Computational Methods and Applications" (ed. I. Fernández), 259−283, Elsevier, 2021.

19. Ponec, R., Bučinský, L., Gatti, C. Relativistic Effects on Metal−Metal Bonding: Comparison of the Performance of ECP and Scalar DKH Description on the Picture of Metal−Metal Bonding in $Re_2Cl_8^{2-}$. *J. Chem. Theory Comput.* **6**, 3113–3121 (2010).

20. Glendening, E.D., Landis, C.R., Weinhold, F. NBO7.0: New vistas in localized and delocalized chemical bonding theory. *J. Comput. Chem.* **40**, 2234–2241 (2019).

21. Szczepanik, D.W., Mrozek, J. Probing the interplay between multiplicity and ionicity of the chemical bond. *J. Theor. Comput. Chem.* **10**, 471–482 (2011).

22. Szczepanik, D.W. RunEDDB (version 26-Jun-2021), http://www.eddb.pl/runeddb.

23. Lu, T., Chen, F. Multiwfn: A multifunctional wavefunction analyzer. *J. Comput. Chem.* **33**, 580-592 (2012).
